# The Impact of Dimitrios P. Kontoyiannis on Mucormycosis Research

**DOI:** 10.3390/jof10060382

**Published:** 2024-05-27

**Authors:** Russell E. Lewis

**Affiliations:** Department of Molecular Medicine, University of Padua, 35121 Padova, Italy; russelledward.lewis@unipd.it

## 1. Introduction

Dimitrios P. Kontoyiannis is an infectious diseases physician who has made substantial contributions to the field of medical mycology over the course of his 25-year career. His work includes some of the most highly cited papers in the fields of fungal pathogenesis, antifungal therapeutics, and descriptions of the epidemiology and treatment outcomes of every major opportunistic fungal pathogen afflicting patients with hematological malignancies.

As part of this Special Issue of the *Journal of Fungi*, I have been invited to review a quarter century of Dimitrios Kontoyiannis’ contributions to the study of mucormycosis (Table 1). As a long-time collaborator with Dimitrios, I know the Mucorales has fascinated him and stimulated some of his most innovative and creative research. Therefore, I will attempt to provide a narrative arc of Dimitrios’ journey to studying mucormycosis during his career and in the context of caring for immunocompromised patients. While I cannot do justice to the full spectrum of his work and collaborations, I believe his story can provide encouragement to clinicians at the early stages of their career who have a passion for studying neglected, but difficult-to-treat, pathogens.

## 2. Epidemiological Studies of Mucormycosis

Young infectious diseases physicians interested in a specific rare infection are often advised to “go to where the infections are.” Dimitrios seems to have taken this advice to heart. After graduating summa cum laude from the National Kapodistrian University medical school in Athens, Greece and fulfilling compulsory military service, Dimitrios was encouraged by his mentor, Professor George Samonis, to move to Houston, Texas and work as a clinical research fellow at the Department of Infectious Diseases at The University of Texas M.D. Anderson Cancer Center. Clearly, the move to Texas would be a major cultural shock for the young Greek physician just learning to speak English, but it would also serve as the genesis for a lifelong career of studying fungal diseases.

In the early 1990s, the Infectious Diseases department at M.D. Anderson was still under the leadership of Dr. Gerald Bodey, a giant in infectious diseases whose epidemiological and clinical studies at the dawn of the modern chemotherapy era in the 1960s laid the foundation for empirical antibiotic and antifungal treatment approaches for neutropenic fever [3]. Bodey was one of the first researchers to highlight the significance of undiagnosed opportunistic fungal infections, including mucormycosis, when he reported that Mucorales comprised the third most commonly identified fungal pathogen at autopsy in patients who died with acute leukemia [4].

When he arrived at M.D. Anderson, Dimitrios began working with Elias Anaissie, a hematologist–infectious diseases specialist and a leading investigator in clinical mycology. At that time, the most pressing clinical questions surrounded the use of a recently approved antifungal, fluconazole, and whether it was truly as effective as the “fungicidal” agent amphotericin B for the treatment of invasive candidiasis in neutropenic patients. However, as fluconazole prophylaxis became established as the standard of care, the research focus shifted to breakthrough infections with fluconazole-resistant non-*Candida albicans* yeast and mold infections such as invasive aspergillosis. At that time, mold infections were diagnosed relatively late in the course of infection by culture and had poor response to conventional amphotericin B–deoxycholate, with mortality rates approaching 80% [5].

One of the first papers Dimitrios published in 1994 described three fatal cases of *Cunninghamella bertolletiae* in patients with leukemia and persistent neutropenia [6]. Only two of the infections were diagnosed before patient death. Two of the three patients were treated with an investigational triazole being developed by Schering–Plough (SCH 39304) [7], which was a precursor to (SCH56592), now known as posaconazole, a well-established oral treatment option for mucormycosis [8].

After the completion of his clinical research experience, Dimitrios decided to obtain license as a physician in the United States, which required the completion of US medical exams and an internal medicine residency. Dimitrios matched at the Baylor College of Medicine in Houston and was elected the Chief Resident during his final year of training. Dimitrios then decided to pursue specialization in infectious diseases and was accepted into the Harvard Medical School Infectious Diseases Clinical Fellowship Program at Massachusetts General Hospital. During this formative time in his career, he encountered many great teachers, including another giant in the field of immunocompromised hosts, Robert H. Rubin. Professor Rubin encouraged Dimitrios to apply for a new joint training program between Harvard and the Massachusetts Institute of Technology developed to expose clinicians to basic science research. Dimitrios was selected for the program and assigned to the laboratory of Professor Gerald Fink, a pioneering yeast geneticist and founding member of the Whitehead Institute. Despite never holding a pipette prior to working in Fink’s lab, Dimitrios survived this intense laboratory experience and was able to apply experience in the genetic manipulation of *Saccharomyces* to the studies of azole resistance in pathogenic yeast [9,10,11,12].

By 1998, Dimitrios was recruited back to the M.D. Anderson Cancer Center in Houston as an Assistant Professor at the Department of Infectious Diseases. One of his first papers published after returning was a review of 24 cases of mucormycosis at M.D. Anderson from 1989 to 1998 [13]. This study is a key epidemiological study because it described the clinical presentation and outcomes of mucormycosis immediately prior to the introduction of *Aspergillus*-active triazoles such as voriconazole. In his review, Dimitrios found that mucormycosis frequently presented as a pneumonia indistinguishable from invasive pulmonary aspergillosis. Disseminated infections were observed in 58% of patients with overall survival rates of 25%. Patients without pulmonary involvement who underwent surgical debridement of the sinuses and received prolonged amphotericin B-based therapy with neutrophil recovery had the best prognosis for survival [13].

By the late 1990s, itraconazole capsules or solution were the only feasible oral prophylaxis options for invasive molds. Itraconazole had unpredictable bioavailability, caused GI distress and liver injury, and was associated with a number of severe drug–drug interactions with commonly administered immunosuppressants and chemotherapies. While new lipid formulations of amphotericin B, including one developed at The MD Anderson Cancer Center (amphotericin B lipid complex-Ablecet^®^) were now widely available, dose-limiting nephrotoxicity still limited prolonged treatment courses.

Fortunately, diagnostic capabilities for invasive aspergillosis were improving. *Aspergillus* infections were being detected earlier with widening use of early thoracic computed tomography (CT) and fiberoptic bronchoscopy. By 2004, the Platelia galactomannan ELISA assay for serum was approved by the US Food and Drug Administration for the early diagnosis of aspergillosis, even though some institutions had been previously using unstandardized “homebrew” antigen and PCR-based assays.

The introduction of two new mold-active triazoles-voriconazole (2002) and later posaconazole (2006) were milestones in the treatment of invasive aspergillosis. Both triazoles could be given orally and had fewer toxicities compared to previously available therapies. Although each drug had its own pharmacokinetic, drug interaction, and toxicity problems, *Aspergillus*-attributable mortality began to decrease in many centers with the earlier diagnosis of infections and the availability of more effective treatment options [14,15].

Unfortunately, cases of breakthrough mucormycosis in the literature began to rise after the introduction of voriconazole [16]. In 2005, Dimitrios published a second case-control observational study of mucormycosis at MD Anderson that identified (1) prior voriconazole treatment and (2) breakthrough sinusitis as the two most common clinical clues for Mucorales infection in patients with hematological malignancies [17,18]. Dimitrios’ group also analyzed autopsy records over a 15-year period at MD Anderson that confirmed an increasing prevalence of Mucorales infection post-mortem [19], particularly in later periods after the introduction of voriconazole (2004–2008) [20].

Although voriconazole’s intrinsic lack of activity against Mucorales provided a plausible explanation for the increasing rates of breakthrough infections, Dimitrios hypothesized that prior voriconazole exposure might also possibly modulate the virulence of these more intrinsically resistant fungi. Remarkably, his lab confirmed his hypothesis by showing that an inoculum of *Rhizopus oryzae* grown on voriconazole-containing agar caused significantly more lethal infections in both invertebrate and mammalian models versus inoculum prepared on a drug-free agar [21,22,23]. The same effect was confirmed later for isavuconazole [24] but not posaconazole [25]. To date, the mechanism(s) responsible for this increase in virulence following triazole exposure have not been elucidated, despite recent reports confirming poorer outcomes of breakthrough mucormycosis [26].

In 2005, Dimitrios collaborated with Thomas Walsh, Maureen Roden, and others to publish a comprehensive summary of 929 mucormycosis cases in the literature including clinical presentation by underlying disease, treatments, and outcome [27]. This paper remains the most highly cited paper on Mucorales in humans in the English medical literature (2113 citations at the time of writing, Scopus database www.scopus.com). Dimitrios also led the publication of a frequently cited PATH Alliance registry of mucormycosis cases in patients undergoing solid organ or hematopoietic stem cell transplantation [28].

More recently, Dimitrios has authored a number of useful reviews on more unusual presentations of mucormycosis, including breakthrough infections in patients with hematological malignancies and hyperglycemia [29], osteoarticular infections [30,31], gastrointestinal infections [32], CNS infections [33,34], infection risk after hurricanes and flooding [35], healthcare-associated mucormycosis [36], cutaneous mucormycosis associated with insect bites [37], combat injuries and trauma [38], infections caused by unusual Mucorales [39], and reviews of mucormycosis infections associated with the COVID-19 pandemic [40,41,42].

Dimitrios led the Transnet-Associated Infection Surveillance Network; TRANSNET studies for non-aspergillus mold infections, including Mucormycosis, that described the incidence of these infections in US transplant patients [43,44]. Finally, Dimitrios analyzed the data and also examined the prevalence and overall economic burden of mucormycosis-related hospitalizations in the United States using the Premier Perspective database [45]. With industry and economic colleagues, he found that mucormycosis accounted for 0.12–0.16 of hospitalizations per 10,000 discharges, with a median length of stay of 17 days, 30–37% hospital readmission rate, and average cost per hospital stay of 112,419 US dollars. This study emphasizes the outsized morbidity and costs of the relatively rare fungal infection in US hospitals.

## 3. Diagnostic Studies of Mucormycosis 

An early diagnosis of pulmonary mucormycosis is often missed because of the non-specific radiological findings and lack of molecular (non-culture based) diagnostic tests. In 2005, Dimitrios’ group published a key paper examining the clinical and radiological predictors that favored the diagnosis of pulmonary mucormycosis versus aspergillosis in patients with hematological malignancies [46]. Their findings demonstrated that patients with pulmonary mucormycosis were more likely to present with breakthrough infections on voriconazole with concomitant pansinusitis, multiple pulmonary nodules, and large ipsilateral hilar lymphadenopathy. In collaboration with radiology colleagues at MD Anderson, Dimitrios also published highly cited reviews and analyses of the diagnostic utility of the “reverse halo sign” in pulmonary CT imaging for pulmonary mucormycosis [47].

The prognostic impact of treatment delays in pulmonary mucormycosis was also examined by Dimitrios’ group in 2008. [48]. In an analysis of patients with hematological malignancies, they found that failure to initiate an amphotericin B-based regimen with 6 days of first onset of symptoms was associated with a doubling of 4-week (35.1 vs. 66.1%) and 12-week (48.6 vs. 82.9%) mortality. These data defined a short “window of opportunity” for the antifungal treatment of pulmonary mucormycosis and the urgent medical need for better diagnostic tools.

Although histopathology is often required for definitive diagnosis, the culture recovery of Mucorales hyphae from infected tissue is highly variable depending on how tissue is processed. In collaboration with microbiologists at MD Anderson, Dimitrios developed new incubation techniques to improve tissue recovery of Mucorales [49], and then identified clinical factors that often contributed to culture-negative mucormycosis [50]. Dimitrios also worked with Thomas Walsh’s group to validate new PCR-based assays [51] and other novel molecular tools for an early diagnosis [52]. Kontoyiannis and Shelburne also examined the potential of microbiome sequencing for an early diagnosis of mucormycosis in patients with hematological malignancies [53]. Clearly, the development of better diagnostic tools for invasive mucormycosis remains a major unmet medical need, along with new therapeutic options for the infection.

## 4. Experimental In Vitro Studies Animal Models of Mucormycosis

Although animal models of mucormycosis had been described in the literature since the 1950s [54], most models simulated infection in the setting of diabetic ketoacidosis. Dimitrios’ group developed murine invasive pulmonary mucormycosis models that more faithfully recapitulated the pathogenesis of infection neutropenic patients and characterized the pharmacokinetic/pharmacodynamic (PK/PD) relationships of bloodstream concentrations, tissue levels, and Mucorales pathogen elimination for lipid amphotericin B formulations [55], echinocandins [56,57], and triazoles [58]. A key observation of these studies was that the PK/PD parameter predictive of efficacy in experimental aspergillosis, AUC/MIC, also predicted activity in experimental mucormycosis when indexed to the pathogen MIC. Therefore, putative drug exposures required for treating the more intrinsically resistant Mucorales could be better predicted if the pathogen MIC was known.

Dimitrios’ group also developed a unique cutaneous model of mucormycosis in neutropenic mice that allowed for the close monitoring of lesion progression and response to treatment [59]. This model was used to demonstrate synergistic interactions of calcineurin inhibitors with posaconazole therapy in experimental mucormycosis, as calcineurin plays a key role in many pathogen fungi in stress response, filamentous growth, and virulence [59]. Compared to mice treated with posaconazole alone, mice treated with a combination of tacrolimus plus posaconazole exhibited a significantly greater reductions in Mucorales skin lesion size and fungal burden measured by quantitative CPR (qPCR) and tissue chitin concentrations [59].

These animal model work came to complement interesting in vitro work exploring unique vulnerabilities of Mucorales such the inhibition of mitochondria and cytoplasmic or vacuolar membranes by unconventional drugs (colistin) [60], and conventional and experimental (D-enantiomer antimicrobial peptidomimetics) [61] compounds. In addition, his group has shown experimentally that echinocandins, despite their purported lack of in vitro activity against Mucorales, may have beneficial immunomodulatory activity [57].

### Invertebrate Models

By 1996, the description of how the Toll pathway regulates the expression of specific antimicrobial peptides involved in antifungal defense in fruitflies (*Drosophila melanogaster*) stimulated interest in developing novel invertebrate models of invasive fungal diseases [62]. Kontoyiannis’ laboratory was among the first to adapt Drosophila for studies of virulence and antifungal resistance in Aspergillus spp. [63]. However, a subsequent report from his laboratory published in the *Proceedings of the National Academy of Sciences* (*PNAS*) also demonstrated the possibility of using Drosophila as an invertebrate model system for studies of invasive mucormycosis [64]. Similar to human mucormycosis, corticosteroids, increased iron supply, and iron availability through treatment with deferoxamine dramatically increased the pathogenicity of experimentally induced mucormycosis infection in flies [64]. Whole-genome expression profiling in wild-type flies after infection with Rhizopus oryzae versus A. fumigatus identified genes selectively down-regulated by R. oryzae infection involved in pathogen recognition, immune defense, stress response, detoxification, steroid metabolism, or tissue repair, as well as some genes with an unknown function. Notably, antifungal efficacy in the Drosophila model was concordant with antifungal activity observed in mammalian models used for drug screening [64]. Additionally, phagocytosis and killing by innate immune effector cells in Drosophila were impaired in a similar fashion as human phagocytes for the R. oryzae vs. Aspergillus species [64,65]. Finally, the results from fly models predicted the efficacy of novel combination regimens for mucormycosis, such as the synergistic combination of calcineurin inhibitors plus posaconazole for R. oryzae infection [59].

In the following years, Dimitrios’ group employed the Drosophila model to explore a number of unconventional hypotheses such as (1) whether the culture medium composition used to cultivate sporangiospores changed the virulence of Cunninghamella bertholletiae [66]; (2) whether obesity with high glucose levels and treatment with the glucose controlling agent metformin modulate the severity of mucormycosis in flies [67]; (3) whether shear stress to Mucorales spores similar to that observed in natural disasters (e.g., tornadoes, hurricanes, volcanic eruptions) alter the virulence of pathogens in infection models [68]; and (4) whether cholesterol-lowering statins possess antifungal activity against R. oryzae [69]. Collectively, these studies are a sample of the remarkable versatility of the Drosophila model system for exploring unique pathophysiological aspects of infection as hypothesis-generating research for later confirmation in vertebrate infection models.

One limitation of D. melanogaster is that study endpoints are often limited to survival; therefore, it is difficult to appreciate how infection evolves from the time of inoculation to death. Dimitrios’ group and others also developed a complimentary invertebrate model of Mucormycosis in zebrafish that allows for the real-time monitoring of infection through microscopy to explore how mucosal damage from cytotoxic chemotherapy provides a foothold for fungal invasion by R. arrhizus [70,71]. In their model, treatment with the recombinant human epithelial growth factor receptor ligand epigen suppressed epithelial cell extrusion, leading to reduced fungal invasion and significantly enhanced survival in the model. The zebrafish mucormycosis model uniquely demonstrated the potential of augmenting epithelial restorative capacity to attenuate the pathogenic invasion of fungi associated with human disease.

## 5. Clinical Studies of Mucormycosis

Clinical trials in patients with mucormycosis have been a major hurdle given the rarity of the infection, difficulties in establishing early and accurate diagnosis, and multiple confounding comorbidities and the common presence of coinfections [72]. Dimitrios was involved in several of the most important open-label studies or data registries that established the clinical utility of posaconazole [73] and isavuconazole [74] in mucormycosis treatment. His group also reviewed experience from the MD Anderson Cancer Center, demonstrating a lack of benefits for combination therapy with currently available therapies [75]. Dimitrios helped design and complete the only placebo-controlled, double-blind randomized controlled trial performed for mucormycosis that evaluated deferasirox–liposomal amphotericin B therapy for mucormycosis [76]. While the trial ultimately did not find a benefit for deferasirox due to imbalances in the patient population enrolled, it served as a critical guidepost for future clinical trial considerations for this difficult-to-study mycoses. In a practical sense, some of Dimitrios’ most important contributions to the clinical literature on mucormycosis treatment are probably reflected by his authoritative reviews [77] on topics often non-extensively addressed in clinical guidelines, including the adjunctive use of hyperbaric oxygen [78], surgical management, and treatment duration decisions in the setting of long-term drug toxicities, and monitoring treatment and relapse through PET-CT [79,80,81].

## 6. Immunotherapy for Mucormycosis

As an infectious diseases physician caring for patients at a major cancer research center over the last 30 years, Dimitrios has witnessed firsthand the transformational impact of novel targeted therapies and immunotherapeutic approaches in the treatment of hematological malignancies. He has published several highly cited descriptions of the epidemiological and clinical characteristics of fungal infections, including mucormycosis in the era or new targeted (e.g., ibrutinib, venetoclax) [82] and cellular therapies (e.g., chimeric antigen T-cell, CAR-T). However, more recently, Dimitrios’ research program has sought to harness these novel treatment platforms for cancer and adapt them to control invasive mold diseases [83]. The checkpoint inhibition of PD-1/PD-L1 is a form of cancer immunotherapy that has transformed the treatment of melanoma, certain types of renal, and non-small cell lung cancers, as well as Hodgkin lymphoma. The PD-1 (Programmed Death-1) receptor on T cells and its ligand, PD-L1, play a crucial role in the immune system’s ability to regulate itself to avoid attacking normal cells in the body. However, many cancer cells can express PD-L1, which binds to the PD-1 receptors on T cells, effectively “turning off” T cells and preventing them from attacking the cancer cells. Similar immune evasion techniques may occur with progressive invasive mold infections [83,84].

Using a murine model of pulmonary mucormycosis, Dimitrios’ group recently demonstrated that the checkpoint inhibition of the PD-1/PD-L1 pathway improved clinical outcomes of mucormycosis in immunosuppressed mice, even without concomitant antifungal therapy [85]. Dimitrios has also worked with colleagues to develop laboratory protocols for generating activated *R. oryzae*-specific T cells that can be used as an adoptive T-cell therapy in patients with refractory mucormycosis [86]. Longer-term control with immunotherapy alone or combined with novel antifungal therapies could play an important role in the future for the treatment of this infection considering the intrinsically high resistance rates to antifungals and relatively weak antifungal pipeline of promising agents.

## 7. Conclusions

Dimitrios’ career is a testament to his innovation, collaboration, and versatility in tackling some of the most challenging and difficult issues in medical mycology. His extensive research on mucormycosis has not only advanced our understanding of the disease, but also provided many practical insights about how to improve the clinical management of one of the most devesting infectious complications of chemotherapy. His work in the field of mucormycosis is remarkable considering it is matched (and possibly exceeded) by his contributions to the management of other fungal infections, particularly invasive aspergillosis.

Nevertheless, Dimitrios’ greatest contribution to medical mycology is probably the generations of physicians and post-doctorate fellows whom he has trained both at the patient bedside and in his laboratory. Many of these physicians, such as Dimitrios, arrived at MD Anderson as inexperienced immigrants, but left as independent investigators with the skills and enthusiasm to establish their own research programs in medical mycology. Indeed, many of Dimitrios’s past fellows now occupy prestigious positions in academia and research hospitals throughout the world. This legacy of training the next generation is what will drive not only incremental progress, but also medical breakthroughs of the future for devastating infections such as invasive mucormycosis.

## Figures and Tables

**Table 1:** Top 10 authors publishing papers with subject heading or MESH terms relating to mucormycosis from 1998 to 2024. Data were extracted from 6362 records retrieved from the PubMed database in May 2024 searching “mucormycosis” or “zygomycosis”, and extracted using the R packages PubmedR [1] and Bibliometrix [2].

Author (Last Name, First Initials)	Number of Papers
Kontoyiannis, D.P.	112
Ibrahim, A.S.	88
Chakrabarti, A.	80
Walsh, T.J.	72
Cornely, O.A.	55
Lortholary, O.A.	54
Sharma, S.	51
Kumar, S.	49
Rudramurthy, S.M.	45
Agarwal, R.	45
